# Meta-analysis of laparoscopic anterior resection with natural orifice specimen extraction (NOSE-LAR) versus abdominal incision specimen extraction (AISE-LAR) for sigmoid or rectal tumors

**DOI:** 10.1186/s12957-020-01982-w

**Published:** 2020-08-19

**Authors:** Jun He, Hai-Bo Yao, Chang-Jian Wang, Qin-Yan Yang, Jian-Ming Qiu, Jin-Ming Chen, Zhong Shen, Guan-Gen Yang

**Affiliations:** 1 Department of Colorectal Surgery, Hangzhou Third Hospital, Hangzhou 310009 Zhejiang, People’s Republic of China; 2 Departments of Gastroenterology & Pancreatic Surgery, Zhejiang Provincial People’s Hospital, Hangzhou 310014 Zhejiang, People’s Republic of China

**Keywords:** Natural orifice specimen extraction, NOSE, Laparoscopic anterior resection, Rectal tumor, Sigmoid tumor, Meta-analysis

## Abstract

**Background:**

Natural orifice specimen extraction surgery is a novel technique of minimally invasive surgery. The purpose of this study was to compare the safety of laparoscopic anterior resection with natural orifice specimen extraction (NOSE-LAR) and abdominal incision specimen extraction (AISE-LAR) for sigmoid or rectum tumors.

**Methods:**

MEDLINE (PubMed), Embase, CENTRAL (Cochrane Central Register of Controlled Trials), Scopus, and ClinicalTrials databases were systematically searched for related articles up to August 2019. The primary outcomes included postoperative complications (overall postoperative complication, incision-related complication, anastomotic fistula, and severe complication) and pathologic results (lymph nodes harvested, proximal resection margin, and distal resection edge). The statistical analysis was performed on STATA 12.0 software.

**Results:**

Ten studies comprising 1787 patients were used for meta-analysis. Compared with AISE-LAR, NOSE-LAR had more advantages in terms of overall postoperative complication (odds ratio (OR) = 0.65 (95% CI, 0.46 to 0.90; *P* = 0.01)), incision-related complication (OR = 0.13 (95% CI, 0.05 to 0.35; *P* < 0.01)), distal resection edge (weighted mean difference (WMD) = 0.17 cm (95% CI, 0.02 to 0.33 cm; *P* = 0.02)), recovery of gastrointestinal function (WMD = − 0.38 day (95% CI, − 0.70 to − 0.06 day; *P* = 0.02 )), pain scores in postoperative day 1 (WMD = − 1.64 (95% CI, − 2.31 to − 0.98; *P* < 0.01)), additional analgesics usage (OR = 0.21 (95% CI, 0.11 to 0.40; *P* < 0.01)) and hospital stay (WMD = − 0.71 day (95% CI, − 1.10 to − 0.32 day; *P* < 0.01)), while the operation time of NOSE-LAR was prolonged (WMD = 7.4 min (95% CI, 0.17 to 14.64 min; *P* = 0.04)). The anastomotic fistula, severe complication, lymph nodes harvested, proximal resection margin, intraoperative blood loss, and long-term outcomes in NOSE-LAR were comparable with AISE-LAR.

**Conclusions:**

The safety of NOSE-LAR was demonstrated, and it could be an alternative to conventional surgery in laparoscopic anterior resection for sigmoid and rectal tumors. However, further randomized and multi-center trials are required.

## Introduction

Over the past three decades, laparoscopic surgery has evolved incessantly, especially in the field of colorectal surgery. It has been widely accepted by surgeons and patients in light of the better peri-operative outcomes and analogical long-term effectiveness compared with open surgery for colorectal cancers [1–3].

For conventional laparoscopic colorectal operation, a small laparotomy in the abdomen is required for specimen harvested and colorectal anastomosis. Because of the mini-incision, it causes many undesirable outcomes such as incision pain, wound infection, scar, and even incision hernia, and the advantages of laparoscopic surgery are reduced [4–6]. To minimize those drawbacks, a novel surgical variant known as natural orifice specimen extraction (NOSE) surgery, with the features of natural orifice specimen extraction and total intraperitoneal anastomosis, has been introduced and is becoming a hotspot [7–10]. Some studies have reported the oncology and safety outcomes between NOSE surgery and conventional laparoscopic surgery are comparable [10–13]. And the NOSE surgery, therefore, is supposed to have a progress of minimally invasive surgery.

Recently, some meta-analysis studies had compared natural orifice specimen extraction (NOSE) with abdominal incision specimen extraction (AISE) in laparoscopic colorectum resection for the colorectal disease [14, 15]. However, colorectum resection comprises right hemicolectomy, left hemicolectomy, and anterior resection, et al. Procedures among those surgeries were largely different. And the surgical procedures and the excision extension between sigmoid and rectum resection are similar. In addition, several studies compared laparoscopic anterior resection with natural orifice specimen extraction (NOSE-LAR) with abdominal incision specimen extraction (AISE-LAR) for sigmoid or rectum tumors were recently released [16–21]. Hence, we conducted this meta-analysis to evaluate the safety of NOSE-LAR in sigmoid and rectal tumors.

## Methods

### Study design and inclusion criteria

This meta-analysis followed the Preferred Reporting Items for Systematic Reviews and Meta-analysis (PRISMA) statements [22]. The inclusion and selection criteria were determined before starting a literature search. Only when studies, with full-text, on sigmoid or rectal tumors that compared NOSE-LAR and AISE-LAR and reported at least one of the endpoints of focus were retrieved and analyzed. The most comprehensive research was recruited when overlapping researches was conducted by the same team. No language restriction was applied. Conference abstracts, case reports, reviews, robotic surgery, and single-port laparoscopic surgery were not considered.

Selection criteria conformed to the framework of PICO (Participant, Intervention, Comparison, and Outcome). Patients diagnosed with sigmoid or rectal tumors (benign and malignant tumors) requiring surgery were included. Interventions consisted of NOSE-LAR and AISE-LAR. NOSE-LAR was compared with AISE-LAR in all eligible studies. Primary endpoint outcomes were postoperative complications (overall postoperative complication, abdominal incision-related complication, anastomotic leak, and severe complication (Clavien-Dindo classification ≥ III)) and pathologic results (retrieved lymph nodes, proximal resection edge, distal resection edge). Secondary outcomes included operation time, blood loss, pain score (numeric rating scale score), additional analgesics, gastrointestinal function recovery, hospitalization duration, 5-year disease-free survival (DFS), and 5-year overall survival (OS) [23].

### Search strategy

The following databases had been searched up to August 2019: MEDLINE (PubMed), CENTRAL (Cochrane Central Register of Controlled Trials), Embase, Scopus, and ClinicalTrials. For a more accurate search, the following keywords and/or MeSH terms were used: “Sigmoid Neoplasms,” “Rectal Neoplasms,” “Colorectal Neoplasms,” “Laparoscopy,” “natural orifice specimen extraction,” “transvaginal specimen extraction,” “transanal specimen extraction,” and “transrectal specimen extraction.” The specific search strategies among databases existed differences. The search strategy of PubMed was presented in Additional Text 1. Reference articles of the eligible studies were reviewed to find the potentially relevant studies.

### Study selection and quality assessment

Retrieved studies were independently assessed for relevance by 2 reviewers (Chang-Jian Wang and Jin-Ming Chen) by screening the title and abstract. In order to enhance sensitivity, studies were removed only when both reviewers excluded the study. Subsequently, a full-text assessment was performed on the initial screening included studies. The risk of bias was assessed by the Newcastle-Ottawa Scale (NOS, for observational studies), and studies achieving five or more stars were eligible. Cochrane Collaboration’s tool for assessing risk for bias was used for randomized controlled trials [24, 25]. All discrepancies were discussed before a final decision was made.

### Data collection and statistical analysis

Data from the recruited studies were extracted by two reviewers (Chang-Jian Wang and Jin-Ming Chen) and used for further analysis. Outcome values (mean (standard deviation) and median (interquartile range)) were extracted from each study. Considering potential heterogeneity among studies, we pooled the results by using a random-effects model. The weighted mean difference (WMD) and 95% confidence intervals (CIs) were applied for continuous variables, and the odds ratio (OR) and 95% CIs were used for dichotomous variables. The continuous outcomes were adopted the inverse variance method, and dichotomous outcomes were adopted the Mantel–Haenszel statistical method. When a study merely offered the outcomes with median and interquartile range, an estimation based on formulas designed by Hozo et al. was performed [26]. If a study did not provide the hazard ratio (HR) and 95% CIs of 5-year DFS or/and OS, the methods presented by Tierney et al. were used for data extraction from survival curves [27]. The chi-square test and *I*-squared value were used for measuring heterogeneity, and *I*^2^ > 50% (*P* < 0.10) was defined as significant heterogeneity. Sensitivity analyses (based on NOS score ≥ 6 and the sample size of NOSE-LAR group ≥ 30) were conducted to assess the potential source of heterogeneity and the robustness of the results. Publication bias was examined with a funnel plot and Harbord test. *P* < 0.05 was considered statistically significant. The statistical analysis was performed on STATA 12.0 software.

## Results

### Literature selection and characteristics

**Fig. 1:**
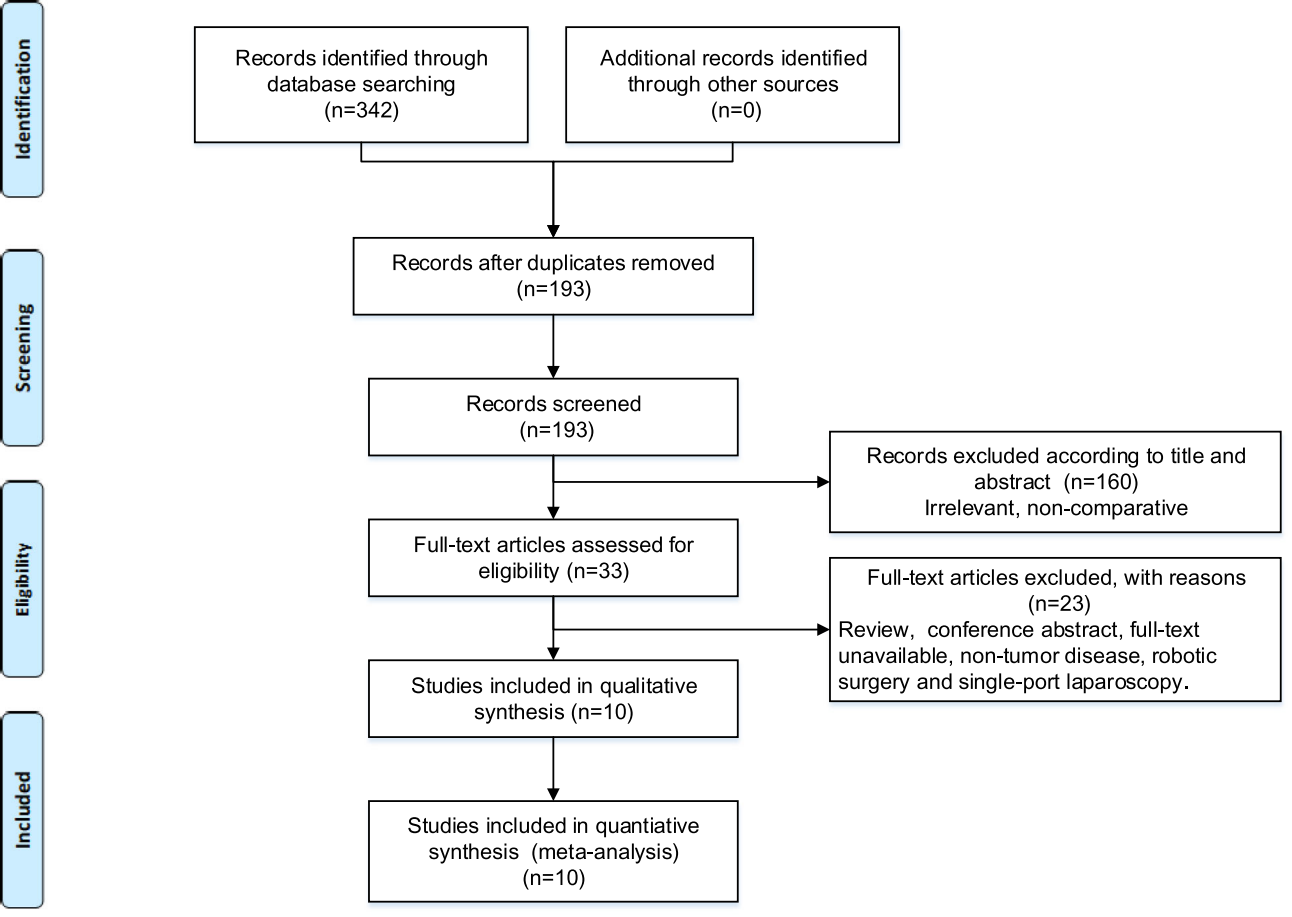
Flow chart of studies included in the meta-analysis

The initial database search identified 342 articles, of which 309 were removed based on the title and abstract assessment. The rest of the literature were evaluated by full-text assessment, and 23 studies were excluded. Characteristics of the excluded studies were presented in Additional Table 1. Ten studies were finally included for further qualitative and quantitative synthesis [11, 13, 15–19, 21, 28, 29]. All of these were retrospective studies. The process of the article search and selection was shown in Fig. 1. A total of 1787 patients were recruited in those studies, with 804 patients in the NOSE-LAR group and 983 patients in the AISE-LAR group. The main characteristics of studies and patients were presented in Table 1, and details were shown in Additional Table 2. The results of the pooled outcomes were summarized in Table 2.

**Table 1:** Main characteristics of the included studies

Study	Year	Region	Study design	Participates (counting)		Age ^a^ (year)		Gender (male/female)		Tumor location	Specimen extraction site	NOS score
				NOSE-LAR group	AISE-LAR group	NOSE-LAR group	AISE-LAR group	NOSE-LAR group	AISE-LAR group			
Hisada et al.	2014	Japan	Retrospective study	20	50	63.7 (9)	66.3 (11)	12/8	NR	Rectum	Anus	5
Hu et al.	2019	China	Retrospective study	26	26	63.1 (8.3)	61.5 (7.6)	17/9	15/11	Rectum	Anus	7
Ng et al.	2018	China	Retrospective study	35	38	65.14 (9.14)	63.95 (9.19)	20/15	22/16	Sigmoid or rectum	Anus	8
Zhang et al.	2014	China	Retrospective study	65	132	56.1 (9.3)	55.5 (9.5)	32/33	57/75	Sigmoid or rectum	Anus	6
Zhou et al.	2019	China	Retrospective study	52	52	55.6 (10.4)	57.0 (10.7)	27/25	27/25	Sigmoid or rectum	Anus	8
Xing et al.	2017	China	Retrospective study	16	32	61.9 (11.8)	62.4 (12.0)	12/4	24/8	Sigmoid	Anus	7
Liu et al.	2019	China, Russia	Retrospective study	356	412	64 (29–79)	62 (32–81)	192/164	235/177	Rectum	Anus or vagina	6
Saurabh et al.	2017	Taiwan	Retrospective study	82	106	63.3 (13.9)	64.7 (10.9)	47/35	65/41	Sigmoid or rectum	Anus	6
Denost et al.	2015	France	Retrospective study	122	98	63 (20–90)	65 (25–85)	70/52	69/29	Rectum	Anus	7
Wang et al.	2019	China	Retrospective study	30	37	58.67 (8.45)	59.70 (10.88)	19/11	20/17	Rectum	Anus	7

Abbreviations: *NOSE-LAR* laparoscopic anterior resection with natural orifice specimen extraction, *AISE-LAR* laparoscopic anterior resection with abdominal incision specimen extraction, *NOS* Newcastle-Ottawa Scale, *NR* not record

^a^Reported as mean ± standard deviation or median (range)

**Table 2:** The pooled results of all outcomes

Outcomes		No. of studies	No. of patients		Pooled results WMD or OR (95%CI)	*P* value	Heterogeneity	
			NOSE-LAR	AISE-LAR			*I*^2 (%)^	*P* value
**Primary outcomes**								
Complication	Overall postoperative complication	10	804	983	0.65 (0.46, 0.90)	0.01	5.0	0.40
	Incision related complication	8	652	848	0.13 (0.05, 0.35)	< 0.01	0	0.98
	Anastomotic fistula	8	647	847	1.09 (0.61, 1.96)	0.78	0	0.81
	Severe complication	2	157	136	0.22 (0.01, 3.66)	0.29	74.1	0.05
Pathologic outcomes	Lymph nodes harvested	9	682	885	− 0.52 (− 1.09, 0.05)	0.07	0	0.64
	Proximal resection margin	3	150	190	0.21 (− 0.73, 1.14)	0.67	0	0.82
	Distal resection edge	4	215	322	0.17 (0.02, 0.33)	0.02	0	0.40
**Secondary outcomes**								
Operation time		9	682	885	7.40 (0.17, 14.64)	0.04	69.9	< 0.01
Intraoperative blood loss		9	682	885	− 10.25 (− 23.22, 2.73)	0.12	89.7	< 0.01
Recovery of gastrointestinal function		6	545	691	− 0.38 (− 0.70, − 0.06)	0.02	89.6	< 0.01
Postoperative pain (POD 1)		4	159	242	− 1.64 (− 2.31, − 0.98)	< 0.01	84.7	< 0.01
Additional analgesics usage		4	159	242	0.21 (0.11, 0.40)	< 0.01	0	0.56
Hospital stay		9	682	885	− 0.71 (− 1.10, − 0.32)	< 0.01	52.5	0.03
Five-year OS		2	174	150	0.69 (0.19, 2.45)	0.56	0	0.92
Five-year DFS		2	174	150	0.83 (0.41, 1.66)	0.59	0	0.55

Abbreviations: *NOSE-LAR* laparoscopic anterior resection with natural orifice specimen extraction, *AISE-LAR* laparoscopic anterior resection with abdominal incision specimen extraction, *WMD* weighted mean difference, *OR* odds ratio, *POD 1* postoperative day 1, *OS* overall survival, *DFS* disease-free survival

### Primary outcomes

**Fig. 2:**
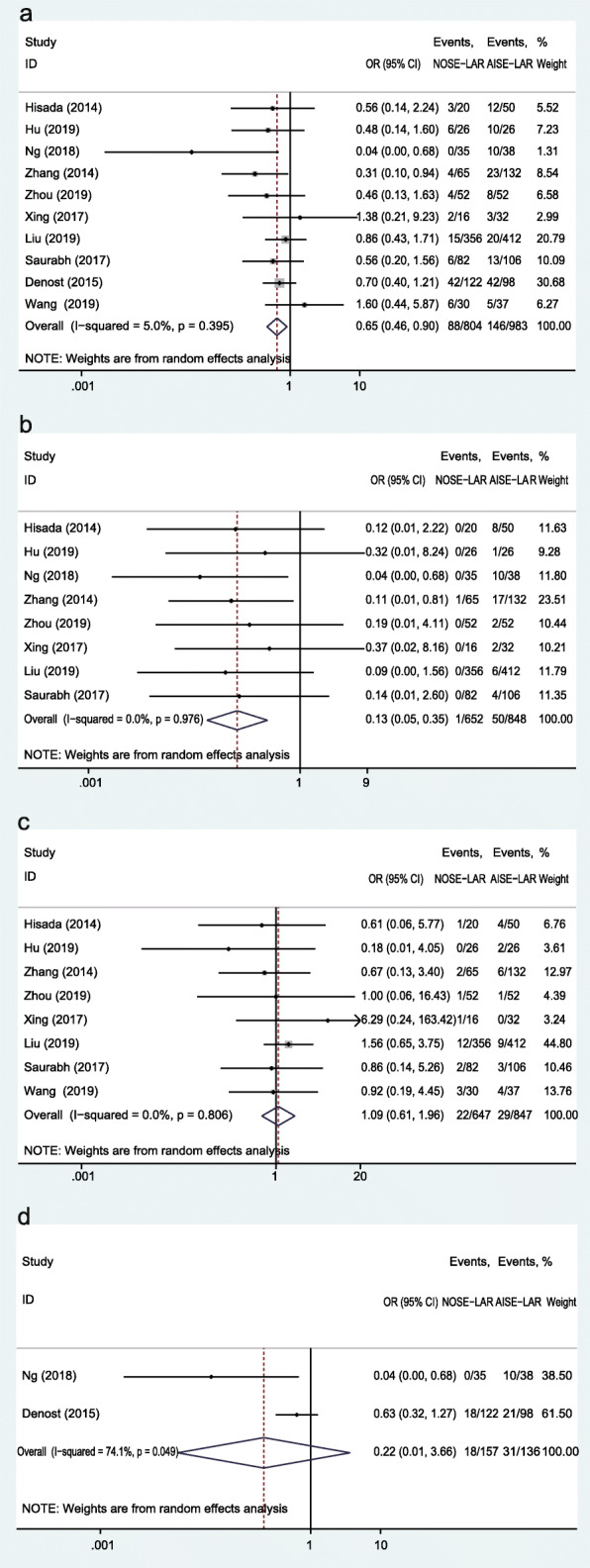
Forest plot comparing postoperative complications in the NOSE-LAR group and AISE-LAR group: **a** overall postoperative complication, **b** incision-related complication, **c** anastomotic fistula, and **d** severe complication

All included studies reported the overall postoperative complication. The pooled data revealed that the postoperative complication in 88 (10.9%) of 804 patients who treated with NOSE-LAR and 146 (14.9%) of 983 patients who treated with AISE-LAR; the OR was 0.65 (95% CI, 0.46 to 0.90; *P* = 0.01) with low heterogeneity (*I*^*2*^ = 5%) (Fig. 2a). Among the 10 studies, eight studies reported the incision-related complication in 1 (0.2%) of 652 patients who underwent NOSE-LAR and 50 (5.9%) of 848 patients who underwent AISE-LAR; the OR was 0.13 (95% CI, 0.05 to 0.35; *P* < 0.01) with no heterogeneity (*I*^*2*^ = 0%) (Fig. 2b). Nine studies reported the anastomotic fistula, of which Ng et al. reported zero events in both groups. The remaining eight studies recorded anastomotic fistula in 22 (3.4%) of 647 patients suffered NOSE-LAR and 29 (3.4%) of 847 patients suffered AISE-LAR; OR was 1.09 (95% CI, 0.61 to 1.96; *P* = 0.78) with no heterogeneity (*I*^*2*^ = 0%) (Fig. 2c). A severe complication was defined based on the Clavien-Dindo classification [30]. Two of the included studies recorded severe complication (Clavien-Dindo classification ≥ III), and the severe complication in 18 (11.5%) of 157 patients with NOSE-LAR and 31 (22.8%) of 136 patients with AISE-LAR; OR was 0.22 (95% CI, 0.01 to 3.66; *P* = 0.29) with significant heterogeneity (*I*^*2*^ = 74%) (Fig. 2d).

**Fig. 3:**
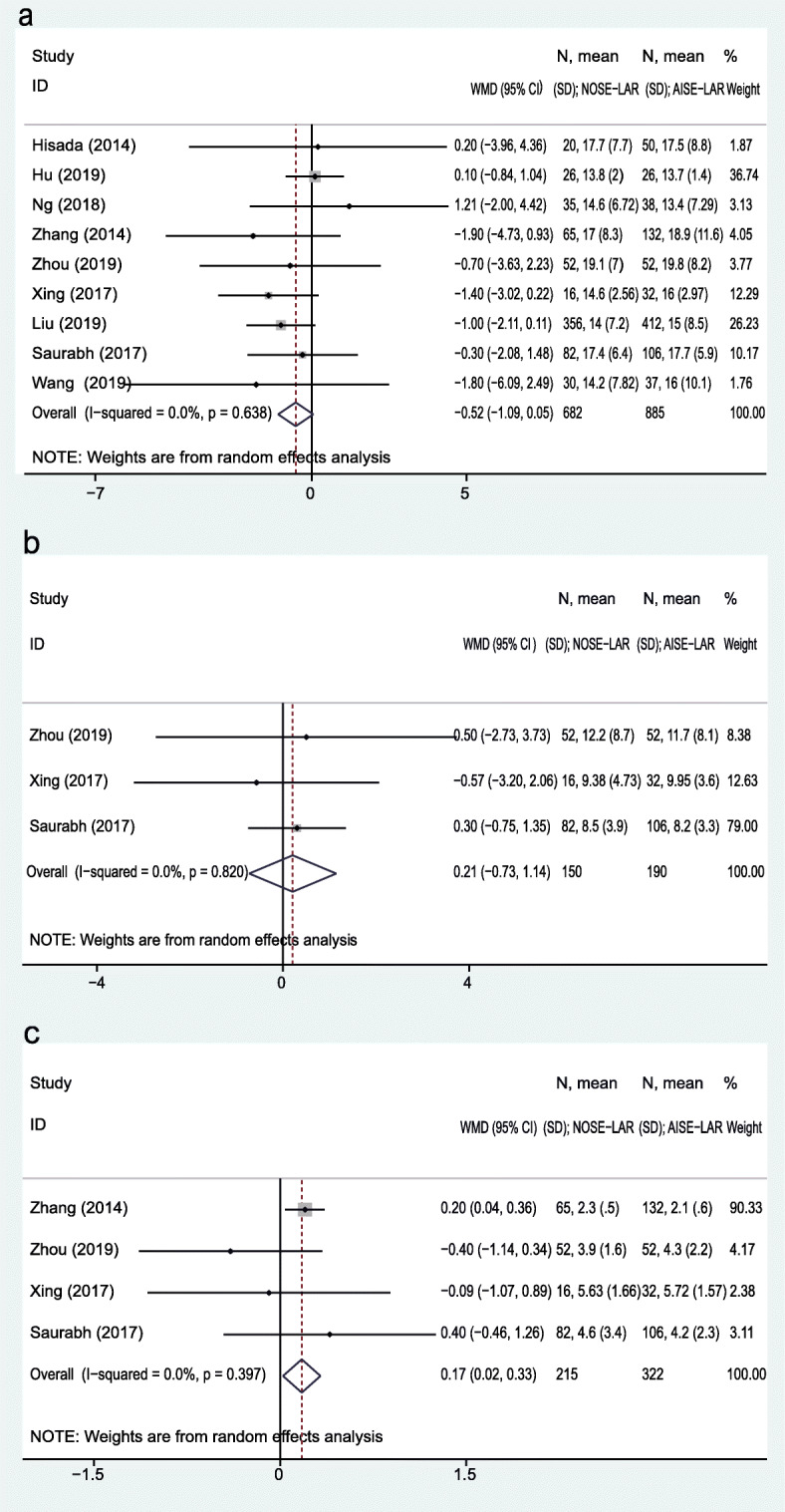
Forest plot comparing pathologic outcomes in the NOSE-LAR group and AISE-LAR group: **a** lymph nodes harvested, **b** proximal resection margin, and **c** distal resection edge

A total of nine studies reported lymph node harvest. There was no significant difference in lymph node harvest between the two groups (WMD = − 0.52; 95% CI, − 1.09 to 0.05; *P* = 0.07). No significant heterogeneity was observed (*I*^*2*^ = 0%) (Fig. 3a). The mean number of dissected lymph nodes in the NOSE-LAR group was 15.2 and the AISE-LAR group was 16.3. The proximal resection margin was reported in 3 studies, and the WMD in the upper resection edge was 0.21 cm (95% CI, − 0.73 to 1.14 cm; *P* = 0.67) with no heterogeneity (*I*^*2*^ = 0%) (Fig. 3b). The distal resection margin was reported in 4 studies, and the WMD in the inferior resection edge was 0.17 cm (95% CI, 0.02 to 0.33 cm; *P* = 0.02) with no heterogeneity (*I*^*2*^ = 0%) (Fig. 3c). The length of the distal resection margin in the two groups was 3.81 cm (NOSE-LAR group) and 3.51 cm (AISE-LAR group).

### Secondary outcomes

**Fig. 4:**
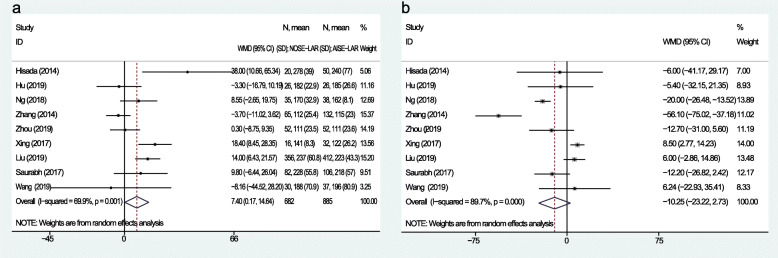
Forest plot comparing intraoperative outcomes in the NOSE-LAR group and AISE-LAR group: **a** operation time and **b** blood loss

A total of nine studies recorded operation time and intraoperative blood loss. The pooled data revealed that the WMD of operative duration was 7.4 min (95% CI, 0.17 to 14.64 min; *P* = 0.04; heterogeneity, *I*^*2*^ = 69.9%) (Fig. 4a). The WMD of blood loss was − 10.25 ml (95% CI, − 23.22 to 2.73 ml; *P* = 0.12; heterogeneity, *I*^*2*^ = 89.7%) (Fig. 4b).

**Fig. 5:**
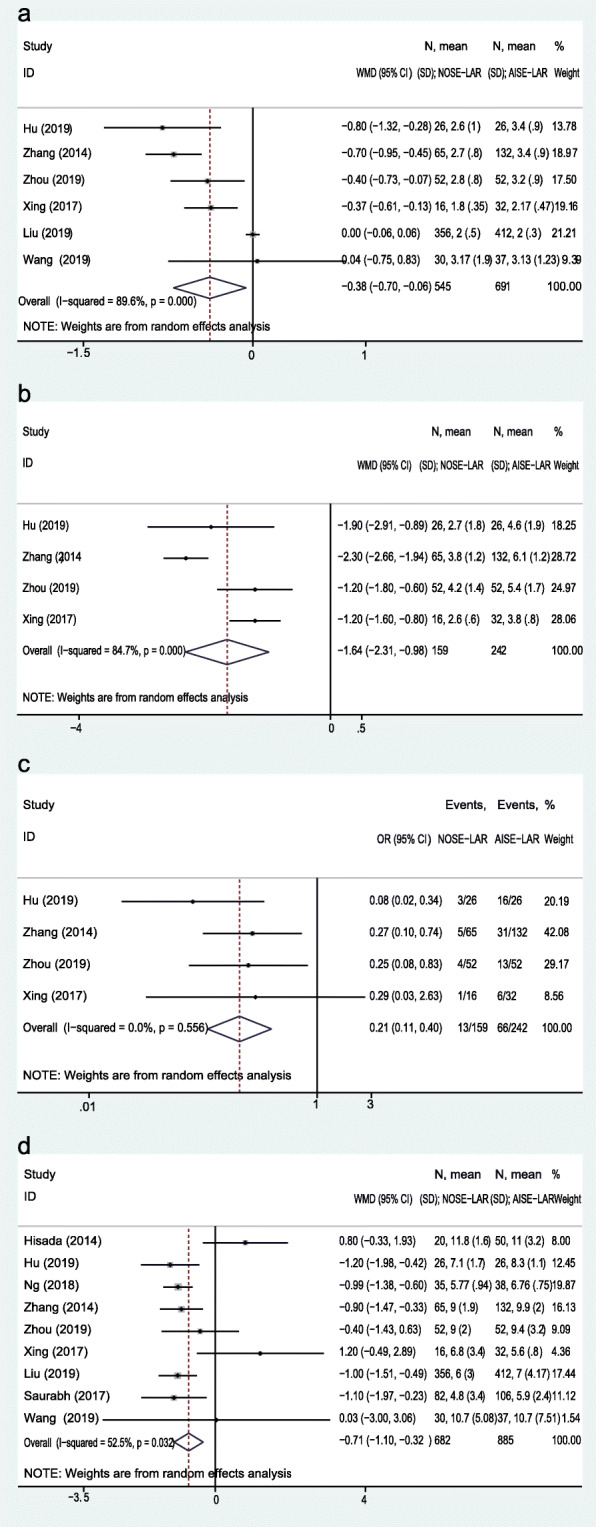
Forest plot comparing postoperative recovery in the NOSE-LAR group and AISE-LAR group: **a** recovery of gastrointestinal function, **b** postoperative pain (POD 1), **c** additional analgesics usage, and **d** hospital stay

Six studies provided data about the recovery of gastrointestinal function. The WMD of bowel movement was − 0.38 day (95% CI, − 0.70 to − 0.06 day; *P* = 0.02; heterogeneity, *I*^*2*^ = 89.6%) (Fig. 5a). The postoperative pain was recorded in 6 studies, and 2 studies (Hisada et al. and Wang et al.) recorded the postoperative pain period and the remaining 4 reported the pain scores in postoperative day 1 (POD 1). The WMD of pain score in POD 1 was − 1.64 (95% CI, − 2.31 to − 0.98; *P* < 0.01; heterogeneity, *I*^*2*^ = 84.7%) (Fig. 5b). The additional analgesic usage rate was also reported in those 4 studies, and the OR of additional analgesics usage was 0.21 (95% CI, 0.11 to 0.40; *P* < 0.01; heterogeneity, *I*^*2*^ = 0%) (Fig. 5c). The duration of hospital stay was reported in nine studies, the WMD of hospital stay was − 0.71 day (95% CI, − 1.10 to − 0.32 day; *P* < 0.01; heterogeneity, *I*^*2*^ = 52.5%) (Fig. 5d).

**Fig. 6:**
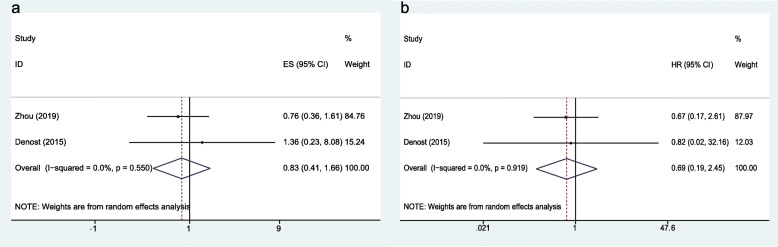
Forest plot comparing long-term outcomes in the NOSE-LAR group and AISE-LAR group: **a** 5-year DFS and **b** 5-year OS

Five-year disease-free survival (DFS) and 5-year overall survival (OS) were available in two studies. The hazard ratio (HR) in the 5-year DFS was 0.83 (95% CI, 0.41 to 1.66; *P* = 0.59); heterogeneity, *I*^*2*^ = 0%) (Fig. 6a), and the HR in the 5-year OS was 0.69 (95% CI, 0.19 to 2.45; *P* = 0.56; heterogeneity, *I*^*2*^ = 0%) (Fig. 6b).

### Sensitivity analysis

Sensitivity analysis results based on the NOS score ≥ 6 and the sample size of NOSE-LAR group ≥ 30 were presented in Additional Table 3. It showed a slight inconsistency in distal resection edge, operation time, and recovery of gastrointestinal function. And all the other outcomes showed a similar trend of results between the two groups.

### Publication bias

**Fig. 7:**
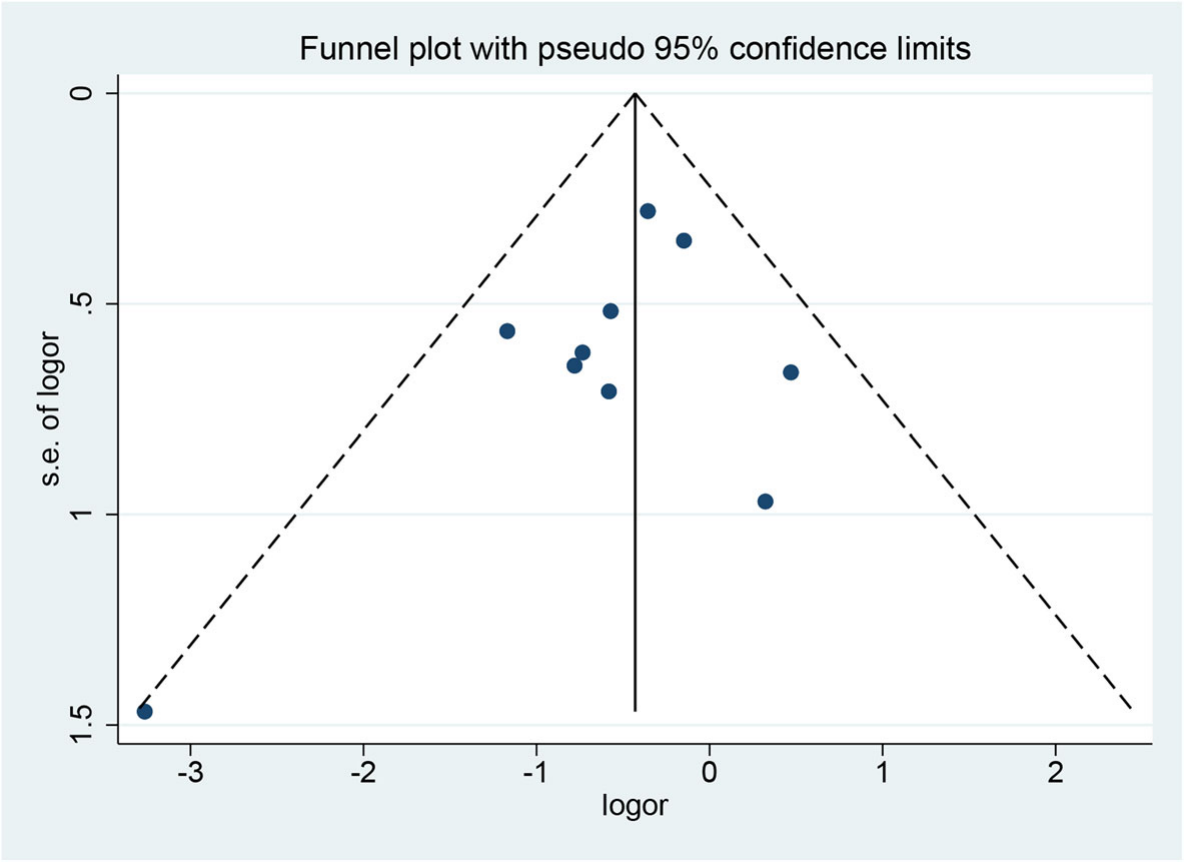
Funnel plot of the overall postoperative complications

A funnel plot of overall postoperative complication was performed to detect publication bias. It showed that all the inclusive studies were within the 95% confidence interval, and no publication bias was found (Fig. 7). In addition, a Harbord test confirmed there was no publication bias (*P* = 0.59).

## Discussion

As a technique used for clinical treatment, the safety of NOSE-LAR should be efficiently proved. Morbidity is one of the most efficient indicators for assessing the safety of an emerging technique. Postoperative complications may not only lead to failures of surgery but also threaten lives. The overall postoperative complication rate in NOSE-LAR (10.9%) was lower than that in AISE-LAR (14.9%). Severe morbidity (Clavien-Dindo ≥ III) among the two techniques was not a significant difference. The operation involving digestive tract reconstruction, anastomotic leakage is a potential risk. Once it occurs, reoperation is usually inevitable [31]. The incidence of anastomotic leakage in NOSE-LAR (3.4%) was similar with that in AISE-LAR (3.4%). In addition, the incidence of incision-related complications in NOSE-LAR (0.2%) was significantly lower than that in the AISE-LAR group (5.9%). Obviously, the reduction of complications in NOSE-LAR has largely attributed to the decrease of incision-related complications. Although the complications in NOSE-LAR were reduced, the risk of bacteria contamination in the peritoneal cavity should not be neglected. Costantino et al. had reported the peritoneal contamination in the NOSE group was higher than that of the conventional group [32]. Hence, measures such as bowel preparation, prophylactic antibiotics, peritoneal irrigation, transanal lavage, transluminal wound retractor, and abdominal drains are recommended to avoid the contamination of the peritoneal cavity [33].

The postoperative pathology results, to some extent, also reflect the safety of a surgery [3]. This meta-analysis showed lymph nodes harvested between the two groups was comparable, and it also conformed to the minimum requirement of the guideline (retrieved more than 12 nodes) [15]. In our meta-analysis, the proximal margin in the NOSE-LAR group was similar with the conventional group. However, the distal margin in the NOSE group was longer than that of the AISE-LAR group. The potential cause of this difference was the use of transanal specimen eversion and extra-abdominal resection technique in the NOSE group [11, 13, 21]. Because of this procedure, the distal rectal resection is performed extra-abdominally under direct vision. Moreover, circumferential resection margin (CRM) between the two groups have no difference [11, 20, 21, 28]. In addition, according to our meta-analysis, the long-term outcomes (5-year DFS and 5-year OS) were comparable. All of those indicated the NOSE technique was a safety procedure in the treatment of sigmoid and rectal cancers. Nevertheless, a concern about tumor seeding was raised during the procedure of enterotomy and specimen extraction. It is necessary to apply several measures such as the use of protection devices (sterile specimen bags) and avoiding over-pulling and compression during specimen extraction [33].

As a minimally invasive surgery, NOSE-LAR had more advantages in alleviating patient’s distress. The reduction of pain scores in postoperative day 1 (POD 1) was observed and this reduction could be attributed to the trauma in NOSE-LAR being further reduced [34]. Owing to less pain, the need for additional analgesics was also reduced. In addition, accelerating postoperative recovery was also observed. The recovery of gastrointestinal function and hospital duration in patients who suffered NOSE-LAR was much shorter. Besides, some scholars may doubt if there have alterations in sexual, urinary, or defecation function in the groups. According to the included studies, there were no differences in functional outcomes such as sexual, urinary, or defecation between two groups [13, 21]. Even though a small part of patients experienced function alteration, and the alternation was reversible [11, 16, 28]. Those all demonstrated that NOSE-LAR was a safety surgery, and to some extent, it had advantages in postoperative recovery.

Nevertheless, our study has several limitations. Firstly, intersphincteric resections were mixed with coloanal anastomoses with sigmoid cancer in our studies. Although there exist some differences, we mixed the two techniques and mainly considered there existing common procedures between sigmoid and rectum resection in laparoscopic anterior resection. And some studies did not record the methods of outcome evaluation (such as blood loss evaluation). To some extent, it reduces the comparability of outcomes. Secondly, the present meta-analysis relied solely on retrospective studies and some original studies not presented how patients were selected to be candidates for one technique or another; the quality of all included studies was regarded as fair or good [35]. However, this type of study cannot be compared with a randomized controlled trial, and potential bias cannot be ruled out. Thirdly, this study only recruited one multicenter research and some outcomes included limited studies. So further multicenter randomized controlled and more comprehensive studies containing adequate outcomes are needed. Fourthly, the results of some pooled results among studies existed heterogeneity. The sensitivity analysis could not be detected as the cause of heterogeneity. Although some results existed heterogeneity, the major results were homogeneity. And the heterogeneity of outcomes such as operation duration, blood loss, and hospital stay can be explained by clinical heterogeneity such as the difference of patients, surgeons, patient management, and differences in surgical proficiency in NOSE technology. In addition, the results of the major parameters were robust. All in all, the results of this analysis are convinced.

According to our meta-analysis, the advantages of NOSE are reduced overall complications (especially incision-related complications), increased distal resection edge, enhanced recovery of gastrointestinal function, reduced postoperative pain, reduced additional analgesics usage, and shortened hospital stay. And without an auxiliary, patients operated by the NOSE technique achieve better aesthetics. However, the operative time is prolonged. Although the NOSE technique has many advantages, there are many requirements that should be followed before the application of this technique in colorectal surgery. Firstly, the NOSE should be operated by experienced surgeons with conventional laparoscopic colorectal surgery. Secondly, the indication of NOSE should follow the indication of conventional laparoscopic colorectal resection. The depth of tumor invasion should be within T3, and body mass index (BMI) should be less than 30 kg/m^2^ for transanal-NOSE and less than 35 kg/m^2^ for transvaginalNOSE. Trans-anal NOSE suits for male or female patients, and the tumor diameter is recommended less than 3 cm. While transvaginal NOSE is only applied for female patients, and the tumor diameter is limited within 5 cm. And the emergent conditions such as bowel obstruction, perforation, and massive bleeding are excluded [33].

## Conclusion

All in all, as surgeons follow appropriate indications, the NOSE-LAR for sigmoid or rectal tumors is a safe surgery. And the long-term outcomes between two operations have no difference, and the benefits of the NOSE-LAR in short-term outcomes are noticeable. These findings promote enthusiasm in support of NOSE surgery as an alternative approach for the treatment of sigmoid and rectal tumors.

## Supplementary information

**Additional file 1.** Additional file Text 1. The search strategy of Pubmed.

**Additional file 2:.** Additional Table 1. Characteristics of the excluded studies.

**Additional file 3.** Additional Table 2. Summary of the included studies.

**Additional file 4.** Additional Table 3. The results of sensitivity analysis.

**Additional file 5.** Additional Table 4. Quality assessment based on the NOS for retrospective studies.

## Data Availability

All the data analyzed in this study was obtained from the included original articles or related authors.

## Abbreviations

NOSE-LAR: Laparoscopic anterior resection with natural orifice specimen extraction
AISE-LAR: Laparoscopic anterior resection with abdominal incision specimen extraction
WMD: Weighted mean difference
OR: Odds ratio
NOS: Newcastle-Ottawa Scale
DFS: Disease-free survival
OS: Overall survival
CIs: Confidence intervals
HR: Hazard ratio
POD 1: Postoperative day 1
