# Supplementary material for: Meta-analysis of laparoscopic anterior resection with natural orifice specimen extraction (NOSE-LAR) versus abdominal incision specimen extraction (AISE-LAR) for sigmoid or rectal tumors
Source: World J Surg Oncol. 2020 Aug 19;18:215. doi: 10.1186/s12957-020-01982-w (PMC7439723; doi:10.1186/s12957-020-01982-w)
Supplement: Supplementary file 2 — Additional file 2:. Additional Table 1. Characteristics of the excluded studies. [file 12957_2020_1982_MOESM2_ESM.doc]

| **Additional Table 1.** **Characteristics of the excluded studies** | | | | |
| --- | --- | --- | --- | --- |
| Study | Year^a^ | Region | Study type | Characteristics or  exclusion reasons |
| Xu et al.[1] | 2016 | China | Retrospective study | Patients containing non-sigomoid or rectum tumor |
| Yin et al.[2] | 2018 | China | Randomized controlled trial | Study ongoing, full-text unavailable |
| Han et al.[3] | 2017 | China | Randomized controlled trial | Study ongoing, full-text unavailable |
| Yu et al.[4] | 2016 | China | Randomized controlled trial | Study ongoing, full-text unavailable |
| Ichiro Takemasa et al.[5] | 2015 | Japan | Prospective non-randomized study | Study ongoing, full-text unavailable |
| Zhou et al.[6] | 2015 | China | Randomized controlled trial | Study ongoing, full-text unavailable |
| Chang et al.[7] | 2018 | China | Retrospective study | Conference abstract, full-text unavailable |
| Chang et al.[8] | 2018 | China | Retrospective study | Conference abstract, full-text unavailable |
| Charnikovskiy et al.[9] | 2018 | Russia | Retrospective study | Conference abstract, full-text unavailable |
| Choi et al.[10] | 2012 | South Korea | Retrospective study | Conference abstract, full-text unavailable |
| Denost et al.[11] | 2012 | France | Retrospective study | Conference abstract, overlap |
| Efetov et al.[12] | 2018 | Russia | Retrospective study | Conference abstract, full-text unavailable |
| Liang et al.[13] | 2012 | United States | Prospective non-randomized study | Conference abstract, full-text unavailable |
| Nakano et al.[14] | 2015 | Japan | Retrospective study | Conference abstract, full-text unavailable |
| Takahashi et al.[15] | 2019 | Japan | Prospective non-randomized study | Conference abstract, full-text unavailable |
| Wang et al.[16] | 2019 | China | Randomized controlled trial | Conference abstract, full-text unavailable |
| Yang et al.[17] | 2016 | South Korea | Retrospective study | Conference abstract, full-text unavailable |
| Zhu et al.[18] | 2019 | China | Retrospective study | Conference abstract, full-text unavailable |
| Kim et al.[19] | 2013 | South Korea | Retrospective study | Containing robotic surgery |
| Kim et al.[20] | 2014 | South Korea | Retrospective study | Containing robotic surgery |
| Kang et al.[21] | 2012 | South Korea | Retrospective study | Containing robotic surgery |
| Park et al.[22] | 2018 | South Korea | Retrospective study | Containing robotic surgery |
| Kim et al.[23] | 2015 | South Korea | Retrospective study | Containing single port surgery |
| ^a^ Publication year or register year | | | | |

**References:**

1. Xu SZ, Ding ZJ, Zhang SF, Qiu XF, Yan F, Yuan SS, Cai JC: **[Propensity score matching-based analysis of short-term outcomes of Laparoscopic-assisted Natural Orifice Specimen Extraction for left colorectal cancer radical resection]**. *Zhonghua Yi Xue Za Zhi* 2016, **96**(20):1578-1581.

2. ChiCTR: **Laparoscopy-assisted natural orifice specimen extraction without incision to treat cancer of the sigmoid colon and rectum: a multicenter prospective randomized controlled trial**. 2018.

3. ChiCTR-IIR-: **Natural orifice specimen extraction versus conventional laparoscopic surgery for radical rectectomy: a prospective, multi-center, randomized controlled trial**. 2017.

4. ChiCTR-INR-: **A randomized controlled clinical study on the treatment of colon cancer with natural orifice and the traditional abdominal cavity**. 2016.

5. JPRN-UMIN: **Prospective Study of Reduced Port Surgery Combined with Transvaginal Specimen Extraction for Colorectal Cancer Resection**. 2015.

6. NCT: **Safety Study of Totally Laparoscopic Resection With Natural Orifice Specimen Extraction (NOSE) for Rectosigmoid Cancer**. 2015.

7. Chang SC, Chen WTL, Wang HM, Ke TW: **Single-incision plus one additional port laparoscopic surgery for colorectal cancer with transanal specimen extraction: A comparative study**. *SURG ENDOSC* 2018, **32**(1):S329.

8. Chang SC, Ke DW, Chung HC, Chen YC, Tsai YY, Hung YC, Chen WT, Wang HM: **3 years oncologic outcomes of transanal specimen extraction after laparoscopic anterior resection for colorectal cancer: A comparative study**. *Surgical Endoscopy* 2018, **32**:S464.

9. Charnikovskiy I, Gavriliukov A, Savanovich N, Smirnov A, Moyseenko V: **LS-surgery of colorectal cancer with transvaginal specimen extraction: Experience of one centre**. *COLORECTAL DIS* 2018, **20**:90.

10. Choi WH, Choi GS, Park JS, Park SY, Kim HJ, Ryuk JP: **Natural orifice specimen extraction versus conventional laparoscopically assisted anterior resection: A case-matched study in 104 patients**. *SURG ENDOSC* 2012, **26**:S243.

11. Denost Q, Gersen H, Celerier B, Capdepont M, Laurent C, Rullier E: **Long term outcome of laparoscopic coloanal anastomosis for low rectal cancer after transabdominal and transanal specimen extraction**. *COLORECTAL DIS* 2012, **14**:8.

12. Efetov S, Kitsenko Y, Milovanova G: **Feasibility of natural orifice specimen extraction (NOSE) surgery for colorectal cancer treatment: Case-matched study**. *COLORECTAL DIS* 2018, **20**:94.

13. Liang S, Franklin JME: **Transanal versus transabdominal specimen extraction with laparoscopic low anterior resection: A comparative analysis on 432 patients with rectal cancer**. *SURG ENDOSC* 2012, **26**:S190.

14. Nakano M, Nishimura A, Kawahara M, Tajima Y: **Totally laparoscopic colectomy with transvaginal specimen extraction versus conventional laparoscopically assisted colectomy**. *SURG ENDOSC* 2015, **29**:S61.

15. Takahashi H, Hamabe A, Hata T, Nishizawa Y, Nishimura A, Itoh M, Takemasa I: **Prospective multicenter study of reduced port surgery combined with transvaginal specimen extraction for colorectal cancer resection**. *Surgical Endoscopy* 2019, **33**:S273.

16. Wang K, Zhu Z, Fu C, Gao W: **Comparison of short-term clinical efficacy and quality of life after transrectal natural orifice specimen extraction, mini-laparotomy, and traditional open surgery for the treatment of colorectal cancer**. *Diseases of the Colon and Rectum* 2019, **62**(6):e240-e241.

17. Yang CS, Choi GS, Park JS, Park SY, Kim HJ, Choi JI, Han KS: **Long-term outcomes of natural orifice specimen extraction for colorectal cancer: A case-matched comparison with a propensity score analysis**. *SURG ENDOSC* 2016, **30**:S31.

18. Zhu Z, Wang K, Lu B, Gao W, Fu C: **Short-term safety of T4a upper rectal and sigmoid cancer treated by laparoscopic radical resection by two different procedures: Natural orifice specimen extraction surgery versus conventional assisted incision**. *Diseases of the Colon and Rectum* 2019, **62**(6):e373.

19. Kim HJ, Choi GS, Park JS, Park SY: **Comparison of intracorporeal single-stapled and double-stapled anastomosis in laparoscopic low anterior resection for rectal cancer: a case-control study**. *INT J COLORECTAL DIS* 2013, **28**(1):149-156.

20. Kim HJ, Choi GS, Park JS, Park SY, Ryuk JP, Yoon SH: **Transvaginal specimen extraction versus conventional minilaparotomy after laparoscopic anterior resection for colorectal cancer: mid-term results of a case-matched study**. *SURG ENDOSC* 2014, **28**(8):2342-2348.

21. Kang J, Min BS, Hur H, Kim NK, Lee KY: **Transanal specimen extraction in robotic rectal cancer surgery**. *Br J Surg* 2012, **99**(1):133-136.

22. Park JS, Kang H, Park SY, Kim HJ, Lee IT, Choi GS: **Long-term outcomes after Natural Orifice Specimen Extraction versus conventional laparoscopy-assisted surgery for rectal cancer: a matched case-control study**. *ANN SURG TREAT RES* 2018, **94**(1):26-35.

23. Kim SJ, Choi BJ, Lee SC: **A novel single-port laparoscopic operation for colorectal cancer with transanal specimen extraction: a comparative study**. *BMC SURG* 2015, **15**:10.
