# Supplementary material for: Meta-analysis of laparoscopic anterior resection with natural orifice specimen extraction (NOSE-LAR) versus abdominal incision specimen extraction (AISE-LAR) for sigmoid or rectal tumors
Source: World J Surg Oncol. 2020 Aug 19;18:215. doi: 10.1186/s12957-020-01982-w (PMC7439723; doi:10.1186/s12957-020-01982-w)
Supplement: Supplementary file 3 — Additional file 3. Additional Table 2. Summary of the included studies. [file 12957_2020_1982_MOESM3_ESM.docx]

**Additional Table 2. Summary of the included studies**

| Study | Year | | Region | Study design | Participates (counting) | | Age ^a^ (year) | | Gender (Male/Female) | | BMI ^a^ (kg/m^2^) | | ASA (I-II/III-IV) | | Tumor size ^a^ (cm) | | Tumor location | Specimen extraction site | NOS score |
| --- | --- | --- | --- | --- | --- | --- | --- | --- | --- | --- | --- | --- | --- | --- | --- | --- | --- | --- | --- |
|  |  |  |  |  | NOSE-LAR group | AISE-LAR group | NOSE-LAR group | AISE-LAR group | NOSE-LAR group | AISE-LAR group | NOSE-LAR group | AISE-LAR group | NOSE-LAR group | AISE-LAR group | NOSE-LAR group | AISE-LAR group |  |  |  |
| Hisada et al. | 2014 | Japan | | Retrospective study | 20 | 50 | 63.7  (9) | 66.3  (11) | 12/8 | NR | NR | NR | NR | NR | 2.7  (0.9) | 3.8  (1.8) | Rectum | Anus | 5 |
| Hu et al. | 2019 | China | | Retrospective study | 26 | 26 | 63.1  (8.3) | 61.5  (7.6) | 17/9 | 15/11 | 26.5  (4.7) | 26.4  (4.6) | 24/2 | 23/3 | NR | NR | Rectum | Anus | 7 |
| Ng et al. | 2018 | China | | Retrospective study | 35 | 38 | 65.14  (9.14) | 63.95  (9.19) | 20/15 | 22/16 | 22.64  (1.95) | 23.41  (1.60) | NR | NR | 3.05  (0.92) | 3.25  (0.99) | Sigmoid or rectum | Anus | 8 |
| Zhang et al. | 2014 | China | | Retrospective study | 65 | 132 | 56.1  (9.3) | 55.5  (9.5) | 32/33 | 57/75 | 23.7  (2.9) | 23.1  (3.1) | 60/5 | 116/16 | 2.9  (1.5) | 3.7  (1.7) | Sigmoid or rectum | Anus | 6 |
| Zhou et al. | 2019 | China | | Retrospective study | 52 | 52 | 55.6  (10.4) | 57.0  (10.7) | 27/25 | 27/25 | 22.7  (2.7) | 23.1  (2.9) | 50/2 | 49/3 | 3.4  (1.3) | 3.7  (1.0) | Sigmoid or rectum | Anus | 8 |
| Xing et al. | 2017 | China | | Retrospective study | 16 | 32 | 61.9  (11.8) | 62.4  (12.0) | 12/4 | 24/8 | 23.1  (1.2) | 23.9  (1.7) | NR | NR | 3.95  (1.21) | 4.37  (1.52) | Sigmoid | Anus | 7 |
| Liu et al. | 2019 | China,Russia | | Retrospective study | 356 | 412 | 64  (29-79) | 62  (32-81) | 192/164 | 235/177 | 22  (17-31) | 23  (18-32) | 258/98 | 299/113 | 3  (1-7) | 4  (1-9) | Rectum | Anus or vagina | 6 |
| Saurabh et al. | 2017 | Taiwan | | Retrospective study | 82 | 106 | 63.3  (13.9) | 64.7  (10.9) | 47/35 | 65/41 | 24.4  (4.2) | 24.4  (3.2) | 59/23 | 75/31 | 2.9  (1.6) | 3.1  (1.6) | Sigmoid or rectum | Anus | 6 |
| Denost et al. | 2015 | France | | Retrospective study | 122 | 98 | 63  (20-90) | 65  (25-85) | 70/52 | 69/29 | 24.3  (17.3-33.6) | 25.8  (18.8-38.3) | NR | NR | 3.9  (1-10) | 4  (1-15) | Rectum | Anus | 7 |
| Wang et al. | 2019 | China | | Retrospective study | 30 | 37 | 58.67  (8.45) | 59.70  (10.88) | 19/11 | 20/17 | 22.65  (3.30) | 22.36  (3.91) | 25/5 | 29/8 | 3.41  (1.40) | 4.24  (1.95) | Rectum | Anus | 7 |

Abbreviations: BMI, body mass index; NOSE-LAR, laparoscopic anterior resection with natural orifice specimen extraction; AISE-LAR, laparoscopic anterior resection with abdominal incision specimen extraction;ASA, American Society of Anesthesiologists;

NOS, Newcastle-Ottawa Scale; NR, not record.

^a^ Reported as mean ± standard deviation or median (range)
