# Supplementary material for: Meta-analysis of laparoscopic anterior resection with natural orifice specimen extraction (NOSE-LAR) versus abdominal incision specimen extraction (AISE-LAR) for sigmoid or rectal tumors
Source: World J Surg Oncol. 2020 Aug 19;18:215. doi: 10.1186/s12957-020-01982-w (PMC7439723; doi:10.1186/s12957-020-01982-w)
Supplement: Supplementary file 4 — Additional file 4. Additional Table 3. The results of sensitivity analysis. [file 12957_2020_1982_MOESM4_ESM.docx]

| **Additional Table 3. The result of sensitivity analysis** | | | | | | | |
| --- | --- | --- | --- | --- | --- | --- | --- |
| Outcomes | No. of studies | No. of patients | | Pooled results WMD or OR (95%CI) | *P*-value | Heterogeneity | |
|  |  | NOSE-LAR | AISE-LAR |  |  | *I*2 （%） | *P*-value |
| Overall postoperative complication |  |  |  |  |  |  |  |
| All studies included | 10 | 804 | 983 | 0.65 (0.46, 0.90) | 0.01 | 5.0 | 0.40 |
| Sample size of NOSE-LAR ≥ 30 | 7 | 742 | 875 | 0.63 (0.40, 0.98) | 0.04 | 30.3 | 0.20 |
| NOS score ≥ 6 | 9 | 784 | 933 | 0.64 (0.44, 0.93) | 0.02 | 15.2 | 0.31 |
| Severe complication |  |  |  |  |  |  |  |
| All studies included | 2 | 157 | 136 | 0.22 (0.01, 3.66) | 0.29 | 74.1 | 0.05 |
| Sample size of NOSE-LAR ≥ 30 | 2 | 157 | 136 | 0.22 (0.01, 3.66) | 0.29 | 74.1 | 0.05 |
| NOS score ≥ 6 | 2 | 157 | 136 | 0.22 (0.01, 3.66) | 0.29 | 74.1 | 0.05 |
| Incision related complication |  |  |  |  |  |  |  |
| All studies included | 8 | 652 | 848 | 0.13 (0.05, 0.35) | < 0.01 | 0 | 0.98 |
| Sample size of NOSE-LAR ≥ 30 | 5 | 590 | 740 | 0.10 (0.03, 0.32) | < 0.01 | 0 | 0.96 |
| NOS score ≥ 6 | 7 | 632 | 798 | 0.13 (0.05, 0.37) | < 0.01 | 0 | 0.95 |
| Anastomotic fistula |  |  |  |  |  |  |  |
| All studies included | 8 | 647 | 847 | 1.09 (0.61, 1.96) | 0.78 | 0 | 0.81 |
| Sample size of NOSE-LAR ≥ 30 | 5 | 585 | 739 | 1.15 (0.61, 2.16) | 0.67 | 0 | 0.90 |
| NOS score ≥ 6 | 7 | 627 | 797 | 1.13 (0.62, 2.08) | 0.68 | 0 | 0.75 |
| Lymph nodes harvested |  |  |  |  |  |  |  |
| All studies included | 9 | 682 | 885 | -0.52 (-1.09, 0.05) | 0.07 | 0 | 0.64 |
| Sample size of NOSE-LAR ≥ 30 | 6 | 620 | 777 | -0.79 (-1.61, 0.02) | 0.06 | 0 | 0.74 |
| NOS score ≥ 6 | 8 | 662 | 835 | -0.54 (-1.11, 0.04) | 0.07 | 0 | 0.54 |
| Proximal resection margin |  |  |  |  |  |  |  |
| All studies included | 3 | 150 | 190 | 0.21 (-0.73, 1.14) | 0.67 | 0 | 0.82 |
| Sample size of NOSE-LAR ≥ 30 | 2 | 134 | 158 | 0.32 (-0.68, 1.32) | 0.53 | 0 | 0.91 |
| NOS score ≥ 6 | 3 | 150 | 190 | 0.21 (-0.73, 1.14) | 0.67 | 0 | 0.82 |
| Distal resection edge |  |  |  |  |  |  |  |
| All studies included | 4 | 215 | 322 | 0.17 (0.02, 0.33) | 0.02 | 0 | 0.40 |
| Sample size of NOSE-LAR ≥ 30 | 3 | 199 | 290 | 0.13 (-0.18, 0.45) | 0.41 | 25 | 0.26 |
| NOS score ≥ 6 | 4 | 215 | 322 | 0.17 (0.02, 0.33) | 0.02 | 0 | 0.40 |
| Five-year OS |  |  |  |  |  |  |  |
| All studies included | 2 | 174 | 150 | 0.69 (0.19, 2.45) | 0.56 | 0 | 0.92 |
| Sample size of NOSE-LAR ≥ 30 | NA | NA | NA | NA | NA | NA | NA |
| NOS score ≥ 6 | NA | NA | NA | NA | NA | NA | NA |
| Five-year DFS |  |  |  |  |  |  |  |
| All studies included | 2 | 174 | 150 | 0.83 (0.41, 1.66) | 0.59 | 0 | 0.55 |
| Sample size of NOSE-LAR ≥ 30 | NA | NA | NA | NA | NA | NA | NA |
| NOS score ≥ 6 | NA | NA | NA | NA | NA | NA | NA |
| Operation time |  |  |  |  |  |  |  |
| All studies included | 9 | 682 | 885 | 7.40 (0.17, 14.64) | 0.04 | 69.9 | < 0.01 |
| Sample size of NOSE-LAR ≥ 30 | 6 | 620 | 777 | 4.81 (-2.47, 12.08) | 0.20 | 61.8 | 0.02 |
| NOS score ≥ 6 | 8 | 662 | 835 | 5.82 (-1.02, 12.67) | 0.10 | 67.2 | < 0.01 |
| Intraoperative blood loss |  |  |  |  |  |  |  |
| All studies included | 9 | 682 | 885 | -10.25 (-23.22, 2.73) | 0.12 | 89.7 | < 0.01 |
| Sample size of NOSE-LAR ≥ 30 | 6 | 620 | 777 | 15.03 (-30.71, 0.64) | 0.06 | 88.6 | < 0.01 |
| NOS score ≥ 6 | 8 | 662 | 835 | -10.58 (-24.21, 3.05) | 0.13 | 91.0 | < 0.01 |
| Recovery of gastrointestinal function |  |  |  |  |  |  |  |
| All studies included | 6 | 545 | 691 | -0.38 (-0.70, -0.06) | 0.02 | 89.6 | < 0.01 |
| Sample size of NOSE-LAR ≥ 30 | 4 | 503 | 633 | -0.29 (-0.72, 0.14) | 0.18 | 91.1 | < 0.01 |
| NOS score ≥ 6 | 6 | 545 | 691 | -0.38 (-0.70, -0.06) | 0.02 | 89.6 | < 0.01 |
| Postoperative pain (POD 1) |  |  |  |  |  |  |  |
| All studies included | 4 | 159 | 242 | -1.64 (-2.31, -0.98) | < 0.01 | 84.7 | < 0.01 |
| Sample size of NOSE-LAR ≥ 30 | 2 | 117 | 184 | -1.78 (-2.85, -0.70) | < 0.01 | 89.6 | < 0.01 |
| NOS score ≥ 6 | 4 | 159 | 242 | -1.64 (-2.31, -0.98) | < 0.01 | 84.7 | < 0.01 |
| Additional analgesics usage |  |  |  |  |  |  |  |
| All studies included | 4 | 159 | 242 | 0.21 (0.11, 0.40) | < 0.01 | 0 | 0.56 |
| Sample size of NOSE-LAR ≥ 30 | 2 | 117 | 184 | 0.26 (0.12, 0.56) | < 0.01 | 0 | 0.92 |
| NOS score ≥ 6 | 4 | 159 | 242 | 0.21 (0.11, 0.40) | < 0.01 | 0 | 0.56 |
| Hospital stay |  |  |  |  |  |  |  |
| All studies included | 9 | 682 | 885 | -0.71 (-1.10, -0.32) | < 0.01 | 52.5 | 0.03 |
| Sample size of NOSE-LAR ≥ 30 | 6 | 620 | 777 | -0.94 (-1.19, -0.69) | < 0.01 | 0 | 0.89 |
| NOS score ≥ 6 | 8 | 662 | 835 | -0.90 (-1.18, -0.63) | < 0.01 | 15.7 | 0.31 |
| Abbreviations: NOSE-LAR, laparoscopic anterior resection with natural orifice specimen extraction; AISE-LAR, laparoscopic anterior resection with abdominal incision specimen extraction; WMD, weighted mean difference; OR, odds ratio; NOS, Newcastle-Ottawa Scale; NA, not available, POD 1, postoperative day 1; OS, overall survival; DFS, diseases-free survival. | | | | | | | |
