# Supplementary material for: Meta-analysis of laparoscopic anterior resection with natural orifice specimen extraction (NOSE-LAR) versus abdominal incision specimen extraction (AISE-LAR) for sigmoid or rectal tumors
Source: World J Surg Oncol. 2020 Aug 19;18:215. doi: 10.1186/s12957-020-01982-w (PMC7439723; doi:10.1186/s12957-020-01982-w)
Supplement: Supplementary file 5 — Additional file 5. Additional Table 4. Quality assessment based on the NOS for retrospective studies. [file 12957_2020_1982_MOESM5_ESM.doc]

| **Additional Table 4. Quality assessment based on the NOS for retrospective studies** | | | | | | | | | | |
| --- | --- | --- | --- | --- | --- | --- | --- | --- | --- | --- |
| Author | Selection | | | | Comparability | | Outcome | | | Score |
|  | 1 | 2 | 3 | 4 | 5 | 6 | 7 | 8 | 9 |  |
| Hisada et al. | * | * | * | * |  |  |  | * |  | 5 |
| Hu et al. | * | * | * | * | * | * |  | * |  | 7 |
| Ng et al. | * | * | * | * | * | * |  | * | * | 8 |
| Zhang et al. | * | * | * | * | * | * |  |  |  | 6 |
| Zhou et al. | * | * | * | * | * | * |  | * | * | 8 |
| Xing et al. | * | * | * | * | * | * |  | * |  | 7 |
| Liu et al. | * | * | * | * |  | * |  | * |  | 6 |
| Saurabh et al. | * | * | * | * | * |  |  | * |  | 6 |
| Denost et al. | * | * | * | * | * |  |  | * | * | 7 |
| Wang et al. | * | * | * | * | * | * |  | * |  | 7 |
| Notes: NOS, Newcastle-Ottawa Quality Scale; 1, representativeness of the exposed cohort; 2, Selection of the non exposed cohort; 3, ascertainment of exposure; 4, outcome was not present at start of study; 5, comparability of the most important factor; 6, comparability of second important factor; 7, assessment of outcome; 8, follow-up long enough; 9, adequacy of follow up. | | | | | | | | | | |
|  |  |  |  |  |  |  |  |  |  |  |
|  |  |  |  |  |  |  |  |  |  |  |
